# On Some Points in the Ordinary Process of Inflammation

**Published:** 1891-08-01

**Authors:** 


### THE PRACTITIONER'S MIRROR AND RETROSPECT.

*[The Editor will be glad to receive offers of co-operation and contributions from members of the profession. All letters should be addressed to* THE EDITOR, THE LODGE, PORCHESTER SQUARE, LONDON, W.]

### MEDICINE.

### ON SOME POINTS IN THE ORDINARY PROCESS OF INFLAMMATION.

The theory of inflammation has been said to be the pivot on which all modern pathology hinges, but the whole process is involved in obscurity. If we ask how it is caused or brought about, we are referred by some teachers to a physiological stimulus, by others to micro-organisms, by others again to ptomaines, while some find that a purely chemical action is sufficient to account for the phenomena. Virchow and his school think that the attraction of the tissues for the blood is increased, while others regard the injury or molecular alteration of the vessel walls as the essential part of inflammation. Perhaps the chief interest lies in the question of the migration of the white cells. How are they able, how are they compelled to pass out of the vessels, and what do they accomplish when outside? The causation of pain, heat, swelling, and redness, and the interference with the nutrition and with the circulation of the part affected, are not without importance after we have clearly determined the action of the migratory corpuscles. It was originally supposed that all the round cells or leucocytes found in inflamed tissues were derived from proliferation of the connective tissue cells, but there seems little doubt that most, if not all of them, come from the blood by direct migration.

Now, Leber has conducted an immense series of experiments which both traverse the ground examined by his predecessors and explore several new regions.* His work has been carried out chiefly by investigations on the action of inflammation in the cornea where blood vessels do not exist. The transparency of the tissue is another advantage, rendering many of the changes visible to the naked eye. After the injection of a fungus, *aspergillus fumigatus*, he observed two distinct changes. At the point of introduction necrosis makes its appearance, while at more distant parts there is simple inflammation, and a special zone of suppuration which gradually invades even the central area. This zone presents a great host of white corpuscles which towards its inner edge are so numerous as to be flattened and squeezed together into a solid mass, while on the outer side they gradually become fewer. The aspergillus in the first few days grows rapidly in the central area, but it is strictly limited by this ring of suppuration. Within it the mycelia spread in every direction, penetrating every layer of the cornea, but none extend beyond the ring. Within it, too, the fixed corneal corpuscles disappear and necrosis takes place, though, at first, after the formation of the ring, there is a space between it and the mycelia which is still transparent. Pus cells from the conjunctiva in small numbers invade the superficial layers of the necrosed portion, and line the membrane of Descemet behind, while they abound in the anterior chamber, and on the surface of the iris. The central area containing the mycelia and the poison they excite is thus surrounded by a wall of cells and in time a groove appears, and the injured tissue is thrown off. The mycelia themselves, in fact, degenerate, and cease to grow after four or five days. In the place, then, where their growth actually took place, there was necrosis, but beyond this area they produced suppuration. He insists that the pus cells are not derived from proliferation of connective tissue corpuscles, nor are those in the anterior chamber derived from the endothelium, where, indeed, the absence of multiplication can be clearly seen. Epithelial and connective cells produce only epithelial and connective tissue cells.

In proof of this he quotes Cohnheim, who injected cinnabar into the dorsal lymph sac of a frog, after causing inflammation in one eye, and found that nearly all the pus cells which accumulated there contained cinnabar.

The mycelium then excretes some poison which, where strongest, causes necrosis, but which, where it is less concentrated, acts on the vessels, producing exudation and an outflow of white corpuscles.

In the same way it was shown that extracts of the aspergillus, staphylococcus aureus, and putrefying muscle produced similar changes, though the microbes themselves were absent, and finally he, Brisger, and others isolated these phlogosins or toxines. But the inflammation excited by these extracts and by certain irritant metals, such as mercury, differs in this, that the process caused by them soon comes to an end, while the living organisms when introduced supply fresh stores of poison, which carry on and extend the destructive changes much longer.

Leber's long-continued experiments present us with a picture of a noxious invader kept within narrow limits and eventually destroyed, while at these limits lies a close array of white corpuscles drawn together from the vessels. It is still open to us to ask whether the corpuscles are the cause of this limitation, or whether, for example, the serofibrinous exudation is the active agent, and the corpuscles mere spectators or victims. To answer this fully would lead us through all the mazes of the phagocyte controversy, but we may point out that the action of the serum has been strongly upheld, notably by Brieger and Kitasato in their researches on tetanus. In fact, a third protective agent is suggested by their method of producing immunity from diphtheria. The microbe may be said to excrete both a poison and antidote. From cultures of some microbes it is possible to isolate a substance protective against future inoculations, and when the circulation is impeded by inflammation, such an excretion may possibly accumulate around Leber's necrotic area and stop the spread of the poison. There are indeed strong arguments for this view. If we accept it, the importance of the inflammatory process would consist in the interference with the circulation of fluids, and not in the action of the corpuscles, or in the bactericide properties of the serum. The action of the white corpuscles on bacteria is only a possible and occasional part of their functions. Whether or no they have the power to limit the spread of such invaders, their migration universally accompanies inflammation, and is of importance from their function of removing effete matters. They may be only helpless victims of bacteria, but they clearly remove other cells. Leber showed that they have the power of liquefying and removing tissues killed by various poisons, and of dissolving gelatine. How they get to the seat of disturbance is also discussed by him with great clearness. He suggests two possible methods. Either they normally wander about in every direction, and those which get within a certain distance of these invaders are paralysed and fixed by the poison, just as we see their amœboid movements are stopped by injurious liquids poured over them. Thus they gradually accumulate around the centre. Or again, the poison, if weak, may act as an irritant to that side of a corpuscle which is nearest to it, increasing the amœboid movements over those on the other side, and thus bringing about motion towards itself. In one way or other an attraction is exercised on the leucocytes which is comparable to that exerted on certain plant cells by

* For an interesting review of Leber's book we may refer to the *Lancet* of June 13th and 27th.

various substances, such as weak acids, and which has been called Chæmotaxis by Pfeffer. That this is not merely a paralytic phenomenon Leber showed by his introduction of fine tubes containing an irritant substance at the closed end into the anterior chamber. These became full of pus, while the eye itself and similarly situated but empty tubes remained free.

These results go a long way towards proving that in inflammation the corpuscles while in the blood stream are themselves attracted, and that the inflammatory changes do not solely affect the vessel walls, as has been said so often to be the case. If the vessels alone were the active cause of the escape of the corpuscles, there would, of course, be no reason for the movements of the corpuscles afterwards, none certainly for their climbing into one tube rather than into another. Whether the exudation of serum and migration of red corpuscles is due to an attraction or to an effect on the vessels Leber gives us no grounds for deciding, nor do we gather any new ideas on the manner in which the actual passage of leucocytes through the walls is effected. So far as the latter are concerned, Leber's experiments seem to us to lead to a position nearer to that maintained by Virchow than Cohnheim's. The process hardly takes place by a change in the filter. The white corpuscles, as Thoma pointed out, unless they retain the power of independent movement, do not escape, and they are affected by a stimulus from without, though this would seem usually to be some diffusible poison, and not the action of the cellular tissues.

